# MUC1 expression and anti-MUC1 serum immune response in head and neck squamous cell carcinoma (HNSCC): a multivariate analysis

**DOI:** 10.1186/1471-2407-6-253

**Published:** 2006-10-25

**Authors:** Martín E Rabassa, María V Croce, Adrián Pereyra, Amada Segal-Eiras

**Affiliations:** 1 Centre of Basic and Applied Immunological Research (CINIBA), Faculty of Medical Sciences, UNLP, Calle 60 y 120, 1900 La Plata, Argentina

## Abstract

**Background:**

HNSCC progression to adjacent tissue and nodes may be mediated by altered glycoproteins and glycolipids such as MUC1 mucin. This report constitutes a detailed statistical study about MUC1 expression and anti-MUC1 immune responses in relation to different clinical and pathological parameters which may be useful to develop new anti HNSCC therapeutic strategies.

**Patients and methods:**

Fifty three pre treatment HNSCC patients were included: 26 (49.1%) bearing oral cavity tumors, 17 (32.1%) localized in the larynx and 10 (18.8%) in the pharynx. Three patients (5.7%) were at stage I, 5 (9.4%) stage II, 15 (28.3%) stage III and 30 (56.6%) at stage IV. MUC1 tumor expression was studied by immunohistochemistry employing two anti-MUC1 antibodies: CT33, anti cytoplasmic tail MUC1 polyclonal antibody (Ab) and C595 anti-peptidic core MUC1 monoclonal antibody. Serum levels of MUC1 and free anti-MUC1 antibodies were detected by ELISA and circulating immune complexes (CIC) by precipitation in polyethylene glycol (PEG) 3.5%; MUC1 isolation from circulating immune complexes was performed by protein A-sepharose CL-4B affinity chromatography followed by SDS-PAGE and Western blot. Statistical analysis consisted in Multivariate Principal Component Analysis (PCA); ANOVA test (Tukey's test) was employed to find differences among groups; nonparametrical correlations (Kendall's Tau) were applied when necessary. Statistical significance was set to p < 0.05 in all cases.

**Results:**

MUC1 cytoplasmic tail was detected in 40/50 (80%) and MUC1 protein core in 9/50 (18%) samples while serum MUC1 levels were elevated in 8/53 (15%) patients. A significant statistical correlation was found between MUC1 serum levels and anti-MUC1 IgG free antibodies, while a negative correlation between MUC1 serum levels and anti-MUC1 IgM free antibodies was found. Circulating immune complexes were elevated in 16/53 (30%) samples and were also statistically associated with advanced tumor stage. MUC1 was identified as an antigenic component of IgG circulating immune complexes. Moreover, poorly differentiated tumors were inversely correlated with tumor and serum MUC1 detection and positively correlated with node involvement and tumor mass.

**Conclusion:**

Possibly, tumor cells produce MUC1 mucin which is liberated to the circulation and captured by IgG antibodies forming MUC1-IgG-CIC. Another interesting conclusion is that poorly differentiated tumors are inversely correlated with tumor and serum MUC1 detection.

## Background

In western countries, head and neck squamous cell carcinoma (HNSCC) occupies the 5th place in frequency and also is the 5th cause of mortality due to cancer. HNSCC localization consists about 40% in the oral cavity, 15% in the pharynx, 25% in the larynx and the rest sites such as salivary glands and thyroid [1,2]. Data obtained from argentinian records show some differences compared with data found in other countries since the most frequent localization has been larynx (1665/3127, 53%) followed by oral cavity (1035/3127, 33%) and finally, pharynx showing 427/3127, 14%. In 2002, the total number of HNSCC cases informed in Argentina was 3127 (16.9/100000) inhabitants [3].

Worldwide, delayed diagnosis is common and frequently very scarce improvement in five-year survival over the last four decades has been observed [4].

HNSCC mainly progress to adjacent tissue and nodes while distant metastasis is a late event. The ability of tumor cells to invade is an acquired and progressive phenomenon mediated, in many cases, by the alteration of membrane glycoproteins such as mucins. Dabelsteen and Gao [5] proposed that the presence of different glycosylation patterns modulate the behavior of these membrane glycoproteins involved in cell signaling. In adenocarcinoma, particular interest has been focused on MUC1 mucin; in previous publications we have extensively detected MUC1 and associated epitopes in HNSCC and also we have isolated this mucin from larynx primary squamous cell carcinoma [6-8]. MUC1 is a large heterodimeric glycoprotein formed by a highly glycosilated extracellular portion associated to a small cytoplasmic tail [9].

Studies developed on different carcinoma localizations such as breast cancer [10,11] have proved that MUC1 mucin can elicit a humoral immune response; furthermore, we have detected free and complexed anti-MUC1 antibodies in serum samples belonging to breast cancer patients [12].

This report constitutes a detailed statistical study about MUC1 expression and anti-MUC1 immune response related to different clinical and pathological parameters which may be useful to increase our knowledge to develop new anti HNSCC therapeutic strategies based on immunological tools.

Here, we present data that confirm a high tumor MUC1 expression in HNSCC which correlates positively with circulating MUC1. Also, a positive correlation was found between serum MUC1 versus anti-MUC1 IgG free antibodies.

In addition, circulating immune complexes levels were statistically associated with tumor size, inversely associated with MUC1 tumor expression and were not positively associated with serum MUC1 and free anti-MUC1 IgG. Finally, tumor size, node involvement and poor differentiation were positively associated.

## Methods

### Patients

Fifty three pre treatment HNSCC patients from the "Hospital General Interzonal de Agudos Gral. San Martín", La Plata, Argentina were included in this study (Table 1); all tumors were primary; patients with previous history of HNSCC or who had received preoperative therapy were not included. Patients were clinically categorized according to the American Joint Cancer Committee (AJCC, Cancer Staging Manual, 2002). Sex and age distribution of patients was: 39 males (73.6%) and 14 (26.4%) females with a mean age of 60.67 (11.55 SD) years and a range from 29 to 98 years. Twenty six patients (49.1%) had their primary tumor located in the oral cavity, 17 (32.0%) in the larynx and 10 (18.9%) in the pharynx. Three patients (5.7%) were at stage I, 5 (9.4%) at stage II, 15 (28.3%) at stage III and 30 (56.6%) at stage IV; stages IVA, IVB and IVC cases where also included. Eighteen were classified as well differentiated (34.6%), 19 as moderately differentiated (36.5%) and 15 as poorly differentiated (28.9%). Patient's data were obtained from clinical records.

Informed consent was obtained in accordance with the Hospital Ethical Commission and the World Medical Association Declaration of Helsinki: Ethical Principles for Medical Research Involving Human Subjects (Helsinki, Finland, 1964 and their modifications).

From each patient, blood and tumor samples were studied; blood samples were obtained before surgery. Serum aliquots were frozen at -70°C until use while tumor samples were immediately fixed in methacarn (60% methanol, 30% chloroform and 10% acetic acid) for two hours and finally placed in 70% ethanol.

### Controls

Eighteen serum samples belonging to normal individuals sex and age matched were included as a negative control group in all assays. Ten newly diagnosed pre treatment patients with systemic lupus erythematosus (SLE) were included as a positive control group for circulating immune complex assays.

### Antibodies (Abs)

The following antibodies were employed: C595, an IgG3 mouse monoclonal antibody directed to de APDTR sequence in the variable number tandem repeats of MUC1 protein core, kindly provided by Dr. A Murray and Dr. S Stevenson (Care of Prof. Alan Perkins) University of Nottingham, UK [13] and CT33, a rabbit polyclonal antibody developed after immunization with a synthetic peptide corresponding to the COOH-terminal 17 amino acids of the intracytoplasmic tail of MUC1, provided by Dr. K. Chul Kim, Lovelace Respiratory Research Institute, Albuquerque, NM, USA [14].

### Immunohistochemistry

Immunohistochemistry was performed according to standard procedures and as previously reported [15]. Surgical specimens were embedded in paraffin, 4 μm sections were cut and placed in bovine serum albumin (BSA) (Sigma, USA) coated slides.

Samples were dewaxed in xylol, rehydrated and endogenous peroxidase activity was blocked by treatment with 0.3% H_2_O_2 _(Merck, Germany) in methanol; slides were incubated in 10% horse normal serum in phosphate buffered saline (PBS) 1% BSA to eliminate background reactions and antigenic recuperation was performed by heating at 100°C in 10 mM sodium citrate buffer.

Slides were incubated overnight with the primary Ab together with negative controls with PBS at 4°C; after incubation with secondary peroxidase labeled Ab, reaction was developed with 3-3'diaminobencidine (Sigma, USA) and H_2_O_2 _(Merck, Germany) in PBS.

Finally, sections were counterstained with hematoxylin (Sigma, USA), dehydrated and coverslipped with mounting media. Samples were evaluated under a light microscope and a positive reaction was considered when more than 5% of the neoplasic cells were stained. Staining intensity was scored in a semiquantitative manner [16] and was graded as negative (-), low (+), moderate (++) and strong (+++). Patterns of reaction were classified as linear (plasma membrane), cytoplasmic, mixed (linear and cytoplasmic) and nuclear following other authors [17,18] with some modifications [15].

Three co-authors (AS-E, MVC and MR) performed a blinded analysis of all samples included; Prof. Amada Segal-Eiras is Professor on Pathology (Faculty of Medical Sciences, UNLP, Argentina).

### Precipitation of circulating immune complexes in polyethylene glycol (PEG) 3,5%

One hundred μl serum sample were mixed with 900 μl of borate buffer (50 mM boric acid and 5 mM sodium borate, pH 8.5) and 1000 μl of 7% PEG 6000 (Fluka AG, Switzerland) in borate buffer, incubated overnight at 4°C and centrifuged at 8000 rpm for 30 minutes at 4°C. Precipitates were resuspended in 2 ml of 3.5% PEG and centrifuged at 7883 g for 30 minutes at 4°C. The supernatant was discarded and the pellet was resuspended in 2 ml of 0.1 N sodium hydroxide. Absorbance was read at 280 nm. A cut off point obtained from the negative control group was calculated at 0.229 OD (cut off = 0.171 + 2 SD) [19].

### Isolation of circulating immune complexes by protein A sepharose-CL4B affinity chromatography

One ml of patient serum was employed following the same procedure described above. Circulating immune complexes were resuspended in 1 ml PBS, applied to a 15 × 1 cm protein A sepharose CL4B column (Sigma, USA) and incubated for 30 min at 4°C. Unbound material was eluted with 15 ml of PBS and 1 ml fractions were collected. For circulating immune complexes recovery, 5 ml of 50 mM glycine chloride (pH 2.8) was added to the column, mixed and incubated for 30 minutes at 4°C followed by elution with 10 ml of glycine chloride, collecting 1 ml fractions.

Protein concentration was monitored by absorbance measurement at 280 nm and the protein peak containing fractions were thoroughly dialyzed.

### Identification of tumor antigens in circulating immune complexes by SDS-PAGE and Western blotting

Electrophoretic separation of proteins [20] was performed with discontinuous 4% stacking-10% resolving polyacrylamide gels in reducing conditions employing a miniVE Vertical Electrophoresis System (Amersham Biosciences Corp., USA). Gels were electroblotted into nitrocellulose membranes (Schleicher & Schuell GmbH, Germany), which were blocked with TRIS buffered saline (TBS) 3% BSA at 4°C overnight and after five washes with TBS membranes were incubated with primary antibody at 4°C overnight [21]. Nitrocellulose membranes were washed and incubated with peroxidase conjugated goat anti-mouse IgG or IgM (1:400) in TBS 3% BSA at room temperature for 3 hours. Finally, reaction was developed with 3.3'diaminobencidine (Sigma, USA) and H_2_O_2 _(Merk, Germany) in 10 mM TRIS buffer.

### Serum MUC1 determination

Levels of serum MUC1 were measured employing a commercial enzyme-linked immunosorbent assay (ELISA) following manufacturer instructions. The Cancer Associated Serum Assay (CASA) (Medical Innovations Ltd, Australia) utilizes two anti MUC1 core protein monoclonal antibodies, BC2 (IgG) as catcher and BC3 (IgM) as tracer, both directed to MUC1 peptide epitope APDTR. Reaction was detected by a peroxidase labeled anti-mouse IgM and 2,2'-azino-bis (3-ethylebenzythiazoline-6-sulfonic acid) (ABTS; Sigma, USA). Optical density (OD) was read at 405 nm, MUC1 levels were extrapolated from a standard curve and the cut off employed was 4 U/ml.

### Serum anti-MUC1 antibodies detection

Detection of antibodies reactive with the protein core of MUC1 was performed by an ELISA described by von Mensdorff-Pouilly [22] with some modifications [23]. Briefly, 100 μl/well of 1 μl/ml BSA were dispensed onto 3912 Falcon III flexible PVC 96 wells plates (BD Labware, USA) and dried overnight at 37°C. After washing, 50 μl of a 100mer peptide containing five tandem repeats of the MUC1 core protein (100 μ/ml) and 50 μl of carbodiimide (1-cycloexil-3-[2 morpholinoethyl] carbodiimide metho-p-sulphonate (Sigma, USA) were dispensed in duplicates. For each sample, 2 wells were incubated with carbodiimide-peptide and two other wells were employed as controls containing 100 μl of blocking buffer (1% BSA PBS) at 4°C overnight. After washing, 150 μl of blocking buffer was added and incubated at room temperature for 1 hour. Plates were washed and diluted human serum samples in blocking buffer (1:80 for IgM and 1:40 for IgG determinations) were applied and incubated overnight at 4°C. After washing, 50 μl of peroxidase conjugated goat anti-human IgM 1:400 or IgG 1:600 (Sigma, USA) in PBS Tween-BSA 0.1% were dispensed and incubated for 1 hour at room temperature. Finally, the reaction was developed with 50 μl of freshly prepared ABTS in 0.1 M citric acid and 0.2 M Na_2_PO_4_H (pH 5) with H_2_O_2_. After 1 hour at room temperature in the dark, OD was read at 405 nm. Free anti-MUC1 core antibody levels were calculated as the difference between the averages of carbodiimide-peptide wells and controls. Cut off was 0.340 OD for anti-MUC1 IgM and 0.274 for IgG Abs.

### Statistical analysis

Multivariate Principal Component Analysis was applied to standardized variables and a correlation table was obtained. Variables (vectors) were plotted against the two principal components extracted (Factor 1 in x axis and Factor 2 in y axis). ANOVA test with post hoc comparisons of means (Tukey's test) was employed to find differences among groups; nonparametrical correlations (Kendall's Tau) were applied when necessary. Statistical significance was set to p < 0.05 in all cases; calculations were performed with STATISTICA for Windows, StatSoft, Inc. (1998), Tulsa, OK, USA.

## Results

### MUC1 tumor expression by immunohistochemistry

**Figure 1:**
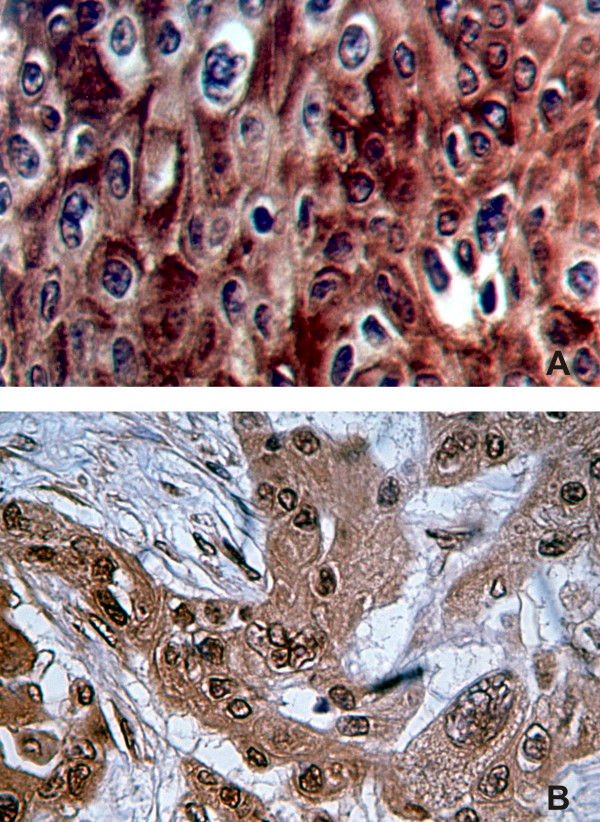
**a **– **Immunohistochemical detection of MUC1 extracellular epitopes**. A stage III larynx carcinoma specimen incubated with anti-MUC1 C595 monoclonal antibody is shown. Cell membrane and cytoplasm are stained over all the picture with a microvesicular pattern. Staining is strong inalmost all cells as well as in high proportion of intercellular connections. **b **– **Immunohistochemical detection of MUC1 cytoplasmic tail epitopes**. Section of a stage IV larynx carcinoma incubated with CT33 antibody is shown. Different cellular patterns are identified. Intense reaction is observed along the cytoplasm with a granular pattern and linear at nuclear membrane. Plasma membrane is partially stained. Cells are polymorphic as well as nuclei, showing different size and vesicular nuclear patterns. Some intercellular bridges are also stained with this antibody.

Tissue MUC1 expression from 50 HNSCC tumor samples was studied employing two different anti MUC1 antibodies (Table 2). CT33 Ab, reactive with MUC1 cytoplasmic tail, was present in 40 (80%) HNSCC specimens, 22/25 tumors localized in oral cavity, 11/16 in the larynx and 7/9 in the pharynx. Reaction pattern was similar to previous reports developed in breast cancer employing the CT2 monoclonal antibody directed against the same last 17 aa of the MUC1 cytoplasmic tail (Croce et al, 2003). Cell membrane staining with a linear pattern was observed in 34 (68%) specimens, a cytoplasmic reaction was obtained in 32 (64%) samples while 27 (54%) had a mixed pattern (Fig. 1b). Also, a nuclear staining was displayed in 4 (8%) specimens. There were no statistically significant differences in MUC1 cytoplasmic tail expression related to tumor stage, localization or differentiation, although a clear tendency of negative CT33 reaction was found in advanced stage tumors while nuclear staining was associated with poorly differentiated tumor areas.

C595 monoclonal antibody, directed to the protein core of MUC1 extracellular domain, showed a positive reaction in 9 samples (18%), 6/25 localized in oral cavity, 2/17 in the larynx and 1/9 in the pharynx. Well differentiated tumors showed a strong reaction limited to the cytoplasm and cell membrane and mainly located in areas with keratin. In moderately and poorly differentiated tumors, a reaction was observed along the cytoplasm with a microvesicular pattern. No correlation was found between C595 monoclonal antibody reactivity versus tumor stage, node status or tumor differentiation. An example of C595 staining is depicted in Fig. 1a.

### MUC1 serum levels

**Figure 2:**
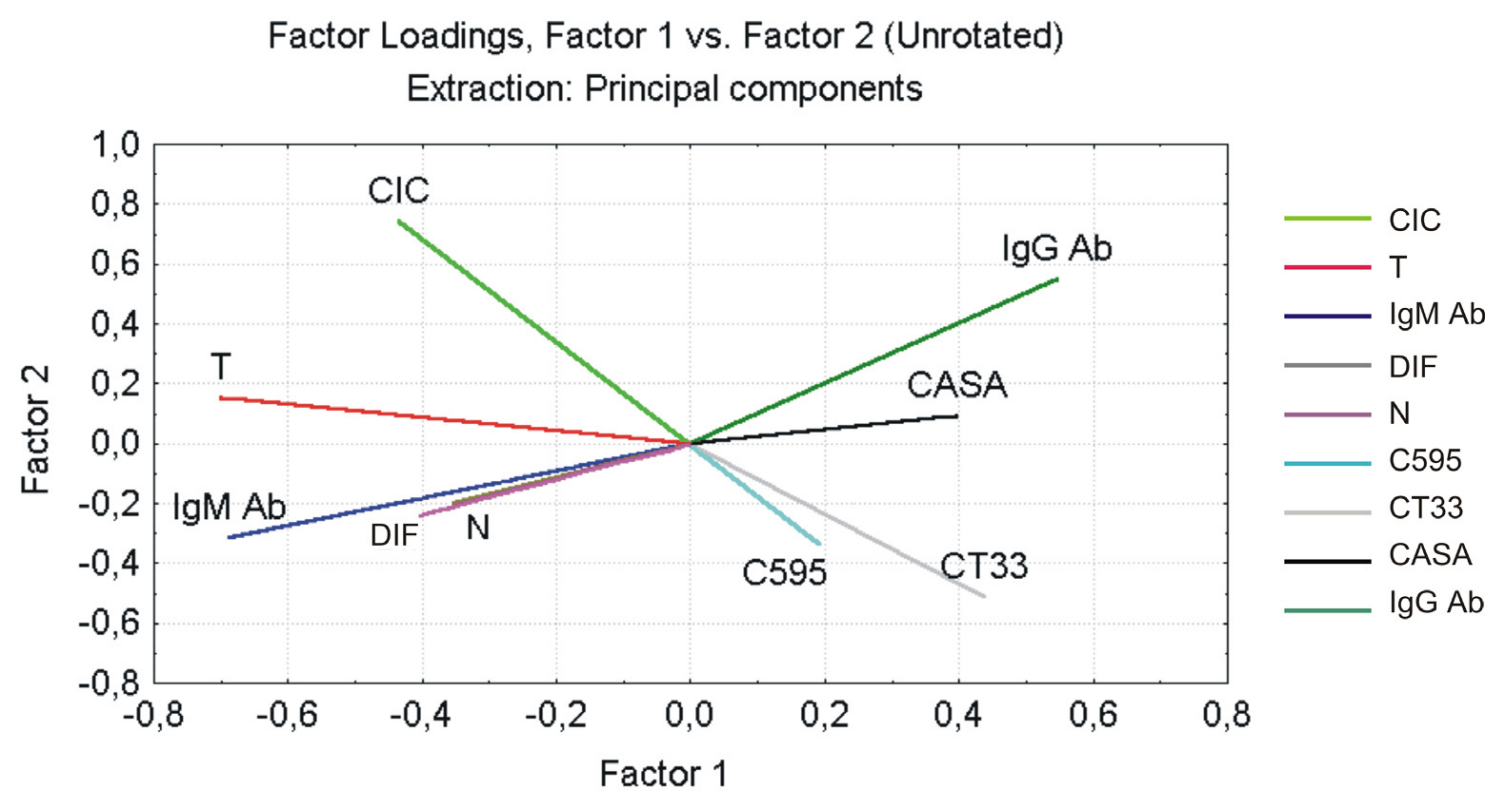
**Multivariate Principal Component Analysis of clinical and histopathological data and tissue and serum expression of MUC1**. Each variable is represented by vectors, which longitude correspond to the weight of the variable in the model, and their projection to both axes corresponds to the weight of the two main factors extracted (Factor 1 and Factor 2). These two factors explain 39% of the variations observedin the model. This percentage is remarkable taking into account that variables belong to patients. CIC = Circulating immune complexes levels; T = Tumor stage; N = Node status; DIF = tumor differentiation; CASA = Serum MUC1 levels; IgM Ab = free anti-MUC1 IgM antibodies; IgG Ab = free anti-MUC1 IgG antibodies; C595 = MUC1 extracellular domain expression; CT33 = MUC1 Cytoplasmic tail expression.

Serum MUC1 levels were measured in 53 HNSCC patients by means of ELISA. MUC1 serum levels were higher in cancer patients (mean = 2.46 U/mL, SD = 3.46 U/mL) than controls (mean = 0.60 U/mL, SD = 0.69 U/mL); the percentage of individuals over the cut off value (4 U/mL) was 8/53 (15%) vs 2/18 (10.5%), respectively. Through multivariate Principal Component Analysis, in HNSCC, a positive correlation was found between immunohistochemistry MUC1 detection and Cancer Associated Serum Assay levels (Kendall τ = 0.275, p = 0.005; Fig. 2).

### Anti-MUC1 humoral immune response analysis

#### Anti-MUC1 Ab detection

**Figure 3:**
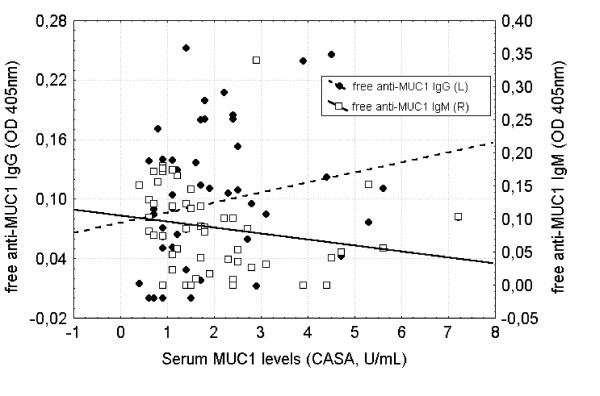
**Correlation between serum MUC1 levels measured by Cancer Associated Serum Assay (U/mL) and free anti-MUC1 antibodies**. Serum MUC1 levels were plotted in abscises against anti-MUC1 IgG Abs (left coordinates) and IgM Abs (right coordinates). A negative correlation between serum MUC1 levels versus anti-MUC1 IgM Abs (τ = -0.266; p < 0.05) and a positive correlation between serum MUC1 levels and IgG Abs are observed (τ = 0.194; p < 0.05).

Free anti-MUC1 serum Ab levels measured in HNSCC were lower than normal sex and age matched controls. Anti-MUC1 IgM mean value in cancer patients was 0.085 OD versus 0.194 OD in controls and anti-MUC1 IgG mean was 0.097 OD vs 0.171 OD. A negative correlation between IgG and IgM levels (Kendall τ = -0.309, p = 0.001) was found. Both anti-MUC1 IgG and anti-MUC1 IgM were statistically correlated with MUC1 serum levels (Fig. 3), while IgG levels were detected according to MUC1 levels, anti-MUC1 IgM levels were very low in those patients over MUC1 cut off levels (4 U/mL). This association was also found by the multivariate Principal Component Analysis; in Fig. 2 the vectors representing anti-MUC1 IgG and IgM are drawn opposed, while the vector that represents serum MUC1 was at the same quadrant of anti-MUC1 IgG.

### Circulating immune complexes in HNSCC patients

**Figure 4:**
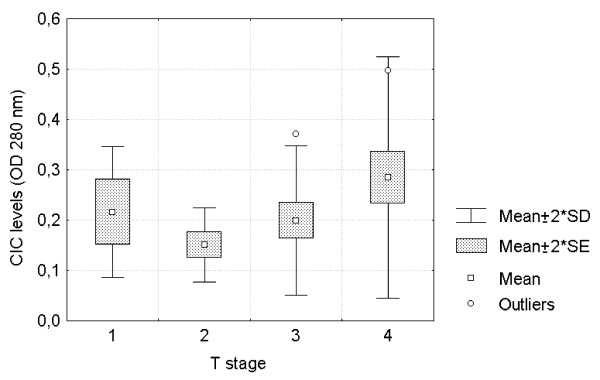
**Comparative analysis of circulating immune complexes' levels (OD 280 nm) among different primary tumor T stages**. Circulating immune complexes' levels in HNSCC increased with tumor T stage (F = 5.375; p < 0.05).

**Figure 5:**
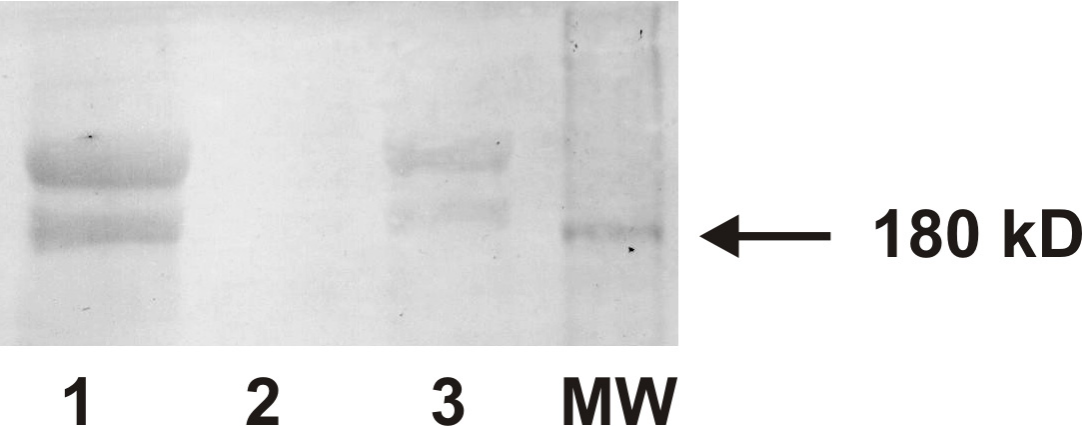
**Western Blot analysis of circulating immune complexes' fractions eluted and isolated from affinity chromatography**. Circulating immune complexes were obtained from a patient with a stage IV larynx tumor. Lane 1: 3.5% PEG precipitate; lane 2: PBS eluate from protein A Sepharose CL-4B column and lane 3: glycine HCl eluate. In lanes 1 and 3 a double band at about 200 kD is observed.

Fifty three HNSCC patients presented a mean circulating immune complexes value of 0.224 OD (SD = 0.103 OD) statistically different from normal negative controls (mean = 0.171 OD, SD = 0.029; p = 0.038) and positive controls (SLE patients), mean = 0.456 OD, SD = 0.121 OD; p < 0.001. Tumor stage was statistically associated with circulating immune complexes levels (Fig. 4); two way ANOVA test with post hoc analysis of means showed differences between T2 and T3 tumors versus T4 although node invasion did not present any difference. Also, a statistical significant difference was found between well differentiated and moderately differentiated tumor samples, which presented a large amount of circulating immune complexes (p < 0.05) (Fig. 5). On the other hand, although undifferentiated tumors had circulating immune complexes levels similar to well differentiated tumors, no statistical difference was found between poor versus moderately differentiated specimens. Elevated circulating immune complexes levels were found in 16/53 patients (30.2%) which belonged to tumor stage I 1/3 (33%), stage III 3/15 (20%) and stage IV 12/30 (40%).

### Identification of tumor antigens isolated from circulating immune complexes

Eight out of 16 (50%) serum samples contained MUC1-CIC; results are shown in Table 3. In each case, clinical data have been included. In Fig. 6, results found in two patients are depicted, Western blot employing C595 monoclonal antibody showed a double band at about 200 kDa. Also, bands at the molecular weight ranging from 25 to 60 kD were observed which correspond to Ig light and heavy chains. No statistical correlation was found between MUC1-CIC and tumor or sera MUC1 expression.

### Principal component analysis correlation studies

Multivariate analysis of MUC1 expression, anti-MUC1 immune response and clinical and pathological parameters in HNSCC showed that histological detection with CT33 and C595 Abs were associated with serum MUC1 levels. Moreover, serum MUC1 levels were positively correlated with anti-MUC1 IgG free antibodies and inversely correlated with anti-MUC1 IgM. This suggested an association between the presence of MUC1 in serum and the type of antibody response.

Vectors representing circulating immune complexes were associated with advanced tumor T stage which may indicate that a large tumor mass produces the MUC1 mucin that reacts with antibodies, forming circulating immune complexes.

Finally, as expected, we found an association between advanced T stage, advanced node status and poor tumor differentiation. Interestingly, the vectors that represent these variables were opposed to serum MUC1 and anti-MUC1 IgG antibody levels.

## Discussion

In our knowledge this is the first report that employs an anti MUC1 cytoplasmic tail Ab in HNSCC; it revealed a high cellular expression of MUC1 (80% of malignant samples).

In previous studies developed in colon and breast cancer [13,14], we have found similar results. The MUC1 cytoplasmic tail amino acid sequence contains seven tyrosine residues constituting intracellular signaling motifs [22] which play important roles in cell response to external stimuli and is related to different oncogenes and genes controlling the cell cycle [23,24][25,26] and β-catenin [27]. Furthermore, it has been reported that in vivo, MUC1 cytoplasmic tail regulates erbB receptors signaling through the activation of MAPK pathways [28].

These observations clearly demonstrate the transforming properties of MUC1 overexpression in carcinoma and emphasize the importance of MUC1 cytoplasmic tail subcellular localization. In HNSCC we have found extensive expression and cellular localization of MUC1 cytoplasmic tail; large and poorly differentiated tumors were likely to be less reactive with CT33 and C595 Abs.

When C595 (anti-extracellular MUC1) monoclonal antibody was assayed, a lower percentage of reactivity with respect to anti-MUC1 cytoplasmic tail was found; we have previously reported a high reactivity to MUC1 protein core epitopes in HNSCC [7,8]. The difference among results was expected since previous reports included a larger number of tumors localized in oral cavity (58%) in comparison with 49.1% in the present report; we found that this localization is reactive with anti-protein core monoclonal antibodies [7]. On the other hand, results presented here corresponded to 18.9% of pharyngeal tumors which did not usually react with these monoclonal antibodies; in past reports, only one patient with a pharyngeal tumor was included [7]. MUC1 cytoplasmic tail reactivity is not affected by MUC1 glycosylation and sialylation and, consequently, represents a better indicator of cell membrane associated MUC1 [15]. Other authors [29-31] have described MUC1 RNA splice variants which do not express the protein core region. Our results also showed that serum MUC1 levels were elevated in 15% of HNSCC samples; interestingly, we found a statistically significant correlation between serum and tumor MUC1 detection indicating that this mucin may possibly be released by the tumor.

In this research we found anti-MUC1 antibodies; also, we observed that serum MUC1 levels were positively correlated with free anti-MUC1 IgG, which may indicate that an anti-MUC1 immune response is mounted. Tumor derived MUC1 is known to induce cellular and humoral specific immune responses in cancer patients [32-34]. In this context, it is possible to speculate that a specific anti-MUC1 immune response is induced in HNSCC patients.

In another report [11], we pointed out that low MUC1 serum levels in stage I breast cancer patients were associated with the presence of free and complexed anti-MUC1 antibodies. Similarly, circulating anti-MUC1-IgG antibody levels were found predictive of survival in breast [35] and pancreatic cancer patients [34]. We have found that anti-MUC1 IgG Ab correlated negatively with poor HNSCC differentiation and disease stage which are the main predictors for patient outcome in this localization.

Furthermore, we proved that MUC1-CIC detected by Western blot were of the IgG isotype since isolation was performed by protein A Sepharose CL-4B chromatography, which is known to bind with high affinity to 1, 2 and 4 IgG classes.

Presence of high amounts of circulating immune complexes in cancer patients is not very well understood. Antibody formation may represent the onset of an anti tumor immune response but, on the other hand, may be the consequence of tumor immune evasion related to a defective cellular immune response. Nonetheless, circulating immune complexes have been associated with tumor progression and also, their formation clearly affects the correct evaluation of several tumor markers.

Only a few reports in the literature have described the antigenic component of circulating immune complexes in HNSCC. Vlock DR and others [36] have exhaustively studied the reactivity of serum Abs against autologous tumor cell lines. These authors found that some patients presented high Ab titers which occasionally cross reacted with tumor cell lines derived from other histogenesis.

In accordance with other reports [37,38], we found higher circulating immune complexes levels in HNSCC patients in comparison with normal subjects; furthermore, we also agree with these authors that elevated circulating immune complexes levels were detected in advanced tumor stage. They pointed out that high circulating immune complexes levels in HNSCC patients is the result of increased tumor mass which would mediate changes in anti tumor immunity. Moreover, Das TK et al [39] found that circulating immune complexes persist after surgical excision of the primary tumor due to the presence of remaining tumor tissue or occult metastasis.

MUC1 is a mucin that has been detected both in the cytoplasm, plasma membrane and nucleus in different tumor localizations. MUC1 is synthesized in the ER and glycosylated in the Golgi apparatus but it has been described that it suffers several glycosylation cycles being expressed in the plasma membrane several times [40]. Furthermore, different splicing variants have been described that can be present in the cytoplasm and in the plasma membrane, as well [31,41-43]. Wen et al [44] investigated intracellular trafficking of MUC1 cytoplasmic tail in human pancreatic cancer cell lines S2-013 and Panc-1 and detected this fraction at the inner cell surface, in the cytosol and in the nucleus. They hypothesized that the association between β-catenin and fragments of the MUC1 cytoplasmic tail facilitated the cytosol-to-nuclear translocation of β-catenin and contributed to its nuclear accumulation. We have found [14] MUC1 cytoplasmic tail and protein core expression in the plasma membrane, cytoplasm and nucleus in breast and colorectal tumor samples.

## Conclusion

In HNSCC, Principal Component Analysis (Fig. 2) showed that circulating immune complexes were associated with advanced tumor stage; furthermore, the vectors that represent these variables did not positively correlate to serum MUC1 and anti-MUC1 IgG antibody levels, and also were inversely correlated with MUC1 tumor expression. Considering that we identified MUC1 as an antigenic component of circulating immune complexes, taken together, these results indicate that a large tumor mass produces high MUC1 mucin that is liberated to circulation and captured by IgG antibodies forming MUC1-CIC.

Another interesting conclusion is that poorly differentiated tumors are inversely correlated with tumor and serum MUC1 detection. This finding suggests the possibility to take into account its consideration regarding prognosis and follow up of this disease.

## Competing interests

The author(s) declare that they have no competing interests.

## Authors' contributions

MER carried out the studies and drafted the manuscript. MVC conceived the study, participated in the design and coordination and wrote the manuscript. AP obtained the samples and clinical records and also participated in the design of the manuscript. ASE participated in the design and coordination of the study and helped to draft the manuscript. All authors read and approved the final manuscript.

## Pre-publication history

The pre-publication history for this paper can be accessed here:



## Figures and Tables

**Table 1:** Patient's clinical data.

Patient Number	Age	Sex	Localization	T stage	N stage	Metastasis	Clinical stage	Tumor differentiation
1	52	M	Larynx	1	0	0	I	Poorly
2	98	M	Larynx	1	0	0	I	Well
3	73	M	Larynx	1	0	0	I	Well
4	57	M	Oral cavity	2	0	0	II	Poorly
5	65	F	Larynx	2	0	0	II	Well
6	68	M	Oral cavity	2	0	0	II	Well
7	64	F	Oral cavity	2	0	0	II	Well
8	63	M	Pharynx	2	0	0	II	Well
9	50	M	Oral cavity	2	1	0	III	Moderately
10	64	M	Larynx	3	1	0	III	Well
11	48	F	Oral cavity	3	1	0	III	Poorly
12	47	F	Oral cavity	3	1	0	III	Well
13	77	M	Oral cavity	3	1	0	III	Poorly
14	59	M	Oral cavity	3	1	0	III	Well
15	69	F	Oral cavity	3	0	0	III	Well
16	56	M	Larynx	3	0	0	III	Moderately
17	65	M	Oral cavity	3	0	0	III	Well
18	40	F	Oral cavity	3	0	0	III	Poorly
19	56	M	Pharynx	3	0	0	III	Poorly
20	70	M	Larynx	3	0	0	III	Moderately
21	75	M	Pharynx	3	0	0	III	Well
22	65	M	Oral cavity	3	1	0	III	Well
23	60	F	Oral cavity	2	1	0	III	Moderately
24	56	M	Pharynx	4	0	0	IVA	Moderately
25	54	F	Pharynx	4	0	0	IVA	Poorly
26	55	M	Oral cavity	4	2	0	IVA	Moderately
27	65	M	Pharynx	4	2	0	IVA	Moderately
28	58	M	Larynx	4	0	0	IVA	Poorly
29	53	M	Larynx	4	0	0	IVA	Moderately
30	62	M	Oral cavity	4	2	0	IVA	ND
31	29	F	Oral cavity	3	2	0	IVA	Moderately
32	60	F	Oral cavity	3	2	0	IVA	Well
33	38	M	Pharynx	4	1	0	IVA	Poorly
34	62	F	Larynx	1	2	0	IVA	Moderately
35	68	M	Larynx	3	2	0	IVA	Moderately
36	68	M	Oral cavity	4	1	0	IVA	Moderately
37	50	M	Oral cavity	3	2	0	IVA	Poorly
38	61	F	Pharynx	2	2	0	IVA	Poorly
39	62	M	Larynx	3	2	0	IVA	Poorly
40	53	M	Larynx	4	2	0	IVA	Well
41	84	F	Larynx	4	0	0	IVA	Well
42	50	M	Oral cavity	4	0	0	IVA	Poorly
43	55	M	Oral cavity	4	0	0	IVA	Moderately
44	73	F	Oral cavity	4	1	0	IVA	Poorly
45	71	M	Oral cavity	4	0	0	IVA	Moderately
46	49	M	Oral cavity	4	0	0	IVA	Well
47	54	M	Oral cavity	4	2	0	IVA	Poorly
48	72	M	Pharynx	4	1	0	IVA	Moderately
49	59	M	Larynx	4	2	0	IVA	Moderately
50	68	M	Larynx	4	3	0	IVB	Moderately
51	54	M	Larynx	3	3	0	IVB	Moderately
52	61	M	Pharynx	4	3	0	IVB	Moderately
53	72	M	Oral cavity	3	3	1	IVC	Well

Site, sex, clinical stage, T and N stage and tumor differentiation are included.

**Table 2:** Immunohistochemical results.

Antibody	Positive/Total	%
C595 (MUC1 Extracellular Domain)	9/50	18
Mixed	4/50	8
Linear	2/50	4
Cytoplasmic	3/50	6
CT33 (MUC1 Cytoplasmic Tail)	40/50	80
Mixed	27/50	54
Linear	34/50	68
Cytoplasmic	32/50	64
Nuclear	4/50	8

Results are expressed as positive cases over total number of cases and percentage, discriminated by immunohistochemical reaction pattern.

**Table 3:** Data from HNSCC patients' serum employed to isolate circulating immune complexes.

Sample	Localization	Stage	Sex	Age	Differentiation	MUC1 In CIC	MUC1 in IHQ	MUC1-CT in IHQ	CIC levels (OD)	MUC1 serum levels (U/mL)
1	Larynx	I	M	52	Poorly	+	-	-	0.306	3.9
2	Larynx	III	M	64	Well	+	-	-	0.371	1.4
3	Larynx	III	M	56	Moderately	+	-	+	0.320	1.2
4	Larynx	III	M	70	Moderately	+	-	+	0.229	0.9
5	Larynx	IVA	M	58	Poorly	-	-	+	0.411	2.2
6	Larynx	IVA	M	53	Moderately	-	-	+	0.312	1.7
7	Oral Cavity	IVA	M	55	Moderately	-	+	+	0.322	4.7
8	Oral Cavity	IVA	F	29	Moderately	+	-	+	0.258	1.7
9	Oral Cavity	IVA	M	68	Moderately	+	-	+	0.323	1.1
10	Oral Cavity	IVA	M	55	Moderately	-	-	-	0.477	0.6
11	Oral Cavity	IVA	M	49	Well	-	-	+	0.403	0.7
12	Pharynx	IVA	M	56	Moderately	-	-	-	0.497	5.3
13	Pharynx	IVA	M	65	Moderately	+	-	+	0.335	1.9
14	Pharynx	IVA	M	38	Poorly	+	+	+	0.231	1.8
15	Pharynx	IVA	M	72	Moderately	-	-	-	0.473	0.4
16	Pharynx	IVB	M	61	Moderately	-	-	+	0.349	1.5
Positive/Total or mean value						8/16	2/16	11/16	0.351	2.0

Clinical data with immunohistochemical detection and serum determination of MUC1, anti-MUC1 Abs and circulating immune complexes are depicted.

## References

[B1] Döbrössy L (2005). Epidemiology of head and neck cancer: magnitude of the problem. Cancer and Metastasis Reviews.

[B2] Shermann CD (1990). Cancer of the head and neck. Manual of Clinical Oncology.

[B3] International Agency for research in Cancer, GLOBOCAN 2002. http://www-dep.iarc.fr.

[B4] Stewart B, Kleihues P, editors (2003). World cancer report.

[B5] Dabelsteen E, Gao S (2005). ABO blood-group antigens in oral cancer. J Dent Res.

[B6] Croce MV, Price MR, Segal-Eiras A (2000). Detection and isolation of MUC1 mucin from larynx squamous cell carcinoma. Pathol Oncol Res.

[B7] Croce MV, Rabassa ME, Price MR, Segal-Eiras A (2001). MUC1 mucin and carbohydrate associated antigens as tumor markers in head and neck squamous cell carcinoma. Pathol Oncol Res.

[B8] Croce MV, Rabassa ME, Pereyra A, Segal-Eiras A (2005). Influence of sialic acid removal on MUC1 antigenic reactivity in head and neck carcinoma. Pathol Oncol Res.

[B9] Taylor-Papadimitriou J, Burchell JM, Plunkett T, Graham R, Correa I, Miles D, Smith M (2002). MUC1 and the immunobiology of cancer. J Mammary Gland Biol Neoplasia.

[B10] Kotera Y, Fontenot JD, Pecher G, Metzgar RS, Finn OJ (1994). Humoral immunity against a tandem repeat epitope of human mucin MUC-1 in sera from breast, pancreatic, and colon cancer patients. Cancer Res.

[B11] von Mensdorff-Pouilly S, Gourevitch MM, Kenemans P, Verstraeten AA, Litvinov SV, van Kamp GJ, Meijer S, Vermorken J, Hilgers J (1996). Humoral immune response to polymorphic epithelial mucin (MUC-1) in patients with benign and malignant breast tumours. Eur J Cancer.

[B12] Croce MV, Isla-Larrain MT, Demichelis SO, Gori JR, Price MR, Segal-Eiras A (2003). Tissue and serum MUC1 mucin detection in breast cancer patients. Breast Cancer Res Treat.

[B13] Price MR, Pugh JA, Hudecz F, Griffiths W, Jacobs E, Symonds IM, Clarke AJ, Chan WC, Baldwin RW (1990). C595 – a monoclonal antibody against the protein core of human urinary epithelial mucin commonly expressed in breast carcinomas. Br J Cancer.

[B14] Croce MV, Isla-Larrain MT, Remes-Lenicov F, Colussi AG, Lacunza E, Kim KC, Gendler SJ, Segal-Eiras A (2006). MUC1 cytoplasmic tail detection using CT33 polyclonal and CT2 monoclonal antibodies in breast and colorectal tissue. Histol Histopathol.

[B15] Croce MV, Isla-Larrain MT, Rua CE, Rabassa ME, Gendler SJ, Segal-Eiras A (2003). Patterns of MUC1 tissue expression defined by an anti-MUC1 cytoplasmic tail monoclonal antibody in breast cancer. J Histochem Cytochem.

[B16] Feickert H, Anger B, Cordón-Cardo C, Lloyd K (1990). Cell-surface antigens of human tumors detected by mouse monoclonal antibodies: definition of blood-group- and non-blood-group-related antigenic systems. Int J Cancer.

[B17] Renkonen J, Paavonen T, Renkonen R (1997). Endothelial and epithelial expression of sialyl Lewis(x) and sialyl Lewis(a) in lesions of breast carcinoma. Int J Cancer.

[B18] Luna-More S, Rius F, Weil B, Jimenez A, Bautista MD, Perez-Mellado A (2001). EMA: a differentiation antigen related to node metastatic capacity of breast carcinomas. Pathol Res Pract.

[B19] Croce MV, Price MR, Segal-Eiras A (1995). Expression of monoclonal-antibody-defined antigens in fractions isolated from human breast carcinomas and patients' serum. Cancer Immunol Immunother.

[B20] Laemmli UK (1970). Cleavage of structural proteins during the assembly of the head of bacteriophage T4. Nature.

[B21] Towbin H, Staehelin T, Gordon J (1979). Electrophoretic transfer of proteins from polyacrylamide gels to nitrocellulose sheets: procedure and some applications. Proc Natl Acad Sci USA.

[B22] Von Mensdorff-Pouilly S, Gouretvich MM, Kenemans P (1989). An enzyme-linked immunosorbent assay for the measurement of circulating antibodies to polymorphic epithelial mucin (MUC1). Tumor Biol.

[B23] Croce MV, Isla-Larrain MT, Capafons A, Price MR, Segal-Eiras A (2001). Humoral immune response induced by the protein core of MUC1 mucin in pregnant and healthy women. Breast Cancer Res Treat.

[B24] Wang H, Lillehoj EP, Kim KC (2003). Identification of four sites of stimulated tyrosine phosphorylation in the MUC1 cytoplasmic tail. Biochem Biophys Res Commun.

[B25] Zrihan-Licht S, Baruch A, Elroy-Stein O, Keydar I, Wreschner DH (1994). Tyrosine phosphorylation of the MUC1 breast cancer membrane proteins. Cytokine receptor-like molecules. FEBS Lett.

[B26] Pandey P, Kharbanda S, Kufe D (1995). Association of the DF3/MUC1 breast cancer antigen with Grb2 and the Sos/Ras exchange protein. Cancer Res.

[B27] Yamamoto M, Bharti A, Li Y, Kufe D (1997). Interaction of the DF3/MUC1 breast carcinoma-associated antigen and beta-catenin in cell adhesion. J Biol Chem.

[B28] Schroeder JA, Thompson MC, Gardner MM, Gendler SJ (2001). Transgenic MUC1 interacts with epidermal growth factor receptor and correlates with mitogen-activated protein kinase activation in the mouse mammary gland. J Biol Chem.

[B29] Wreschner DH, Hareuveni M, Tsarfaty I, Smorodinsky N, Horev J, Zaretsky I, Kotkes P, Weiss M, Lathe R, Dion AS, Keydar I (1990). Human epithelial tumor antigen cDNA sequences – differential splicing may generate multiple protein forms. Eur J Biochem.

[B30] Zrihan-Licht S, Vos HL, Baruch A, Elroy-Stein O, Sagiv D, Keydar I, Hilkens J, Wreschner DH (1994). Characterization and molecular cloning of a novel MUC1 protein, devoid of tandem repeats, expressed in human breast cancer tissue. Eur J Biochem.

[B31] Obermair A, Schmid BC, Stimpfl M, Fasching B, Preyer O, Leodolter S, Crandon AJ, Zeillinger R (2001). Novel MUC1 splice variants are expressed in cervical carcinoma. Gynecol Oncol.

[B32] Agrawal B, Reddish MA, Longenecker BM (1996). In vitro induction of MUC-1 peptide-specific type 1 T lymphocyte and cytotoxic T lymphocyte responses from healthy multiparous donors. J Immunol.

[B33] Petrarca C, Rughetti A, Rahimi H, D'Agostini F, Turchi V, Apollonj Ghetti C, Scambia G, Frati L, Nuti M (1996). Human antibodies against the polymorphic epithelial mucin in ovarian cancer patients recognise a novel sequence in the tandem repeat region. Eur J Cancer.

[B34] Hamanaka Y, Suehiro Y, Fukui M, Shikichi K, Imai K, Hinoda Y (2003). Circulating anti-MUC1 IgG antibodies as a favorable prognostic factor for pancreatic cancer. Int J Cancer.

[B35] von Mensdorff-Pouilly S, Verstraeten AA, Kenemans P, Snijdewint FG, Kok A, Van Kamp GJ, Paul MA, Van Diest PJ, Meijer S, Hilgers J (2000). Survival in early breast cancer patients is favorably influenced by a natural humoral immune response to polymorphic epithelial mucin. J Clin Oncol.

[B36] Vlock DR, Scalise D, Schwartz DR, Richter DE, Krause CJ, Baker SR, Carey TE (1989). Incidence of serum antibody reactivity to autologous head and neck cancer cell lines and augmentation of antibody reactivity following acid dissociation and ultrafiltration. Cancer Res.

[B37] Yamanaka N, Himi T, Harabuchi Y, Hoki K, Kataura A (1988). Soluble immune complexes and squamous cell carcinoma-related antigens in patients with head and neck cancer. Cancer.

[B38] Wolf GT, Wolfe RA (1990). Circulating immune complexes in patients with nasopharyngeal carcinoma. Laryngoscope.

[B39] Das TK, Aziz M, Rattan A, Sherwani R (1995). Prognostic significance of circulating immune complexes in malignant tumours of head and neck. J Indian Med Assoc.

[B40] Litvinov SV, Hilkens J (1993). The epithelial sialomucin, episialin, is sialylated during recycling. J Biol Chem.

[B41] Ligtenberg MJ, Vos HL, Gennissen AM, Hilkens J (1990). Episialin, a carcinoma-associated mucin, is generated by a polymorphic gene encoding splice variants with alternative amino termini. J Biol Chem.

[B42] Williams CJ, Wreschner DH, Tanaka A, Tsarfaty I, Keydar I, Dion AS (1990). Multiple protein forms of the human breast tumor-associated epithelial membrane antigen (EMA) are generated by alternative splicing and induced by hormonal stimulation. Biochem Biophys Res Commun.

[B43] Levitin F, Stern O, Weiss M, Gil-Henn C, Ziv R, Prokocimer Z, Smorodinsky NI, Rubinstein DB, Wreschner DH (2005). The MUC1 SEA module is a self-cleaving domain. J Biol Chem.

[B44] Wen Y, Caffrey TC, Wheelock MJ, Johnson KR, Hollingsworth MA (2003). Nuclear association of the cytoplasmic tail of MUC1 and beta-catenin. J Biol Chem.

